# A Scoping Review of Health Literacy Measurement in U.S. Interventions: Trends, Tools, and Gaps

**DOI:** 10.3928/24748307-20260508-01

**Published:** 2026-10

**Authors:** Yunlin Zhou, Marissa Mathew, Megan S. Patterson, Jason Rosenfeld, Melanie Stone, Tyler Prochnow, Joshua Yudkin

**Affiliations:** a Texas A&M University, College Station; b University of Texas Health San Antonio, San Antonio, Texas.

## Abstract

**Background:**

Health literacy is a critical determinant of health outcomes, yet considerable variability exists in how it is measured across intervention studies. This scoping review article systematically mapped health literacy measurement tools and implementation reporting practices in United States-based interventions published between 2014 and 2024, with particular attention to validated and nonvalidated instruments and their application across diverse populations and settings.

**Methods:**

Following Joanna Briggs Institute guidance and Preferred Reporting Items for Systematic Reviews and Meta-Analyses extension for Scoping Reviews guidelines, we searched Ovid MEDLINE in January 2025 for studies describing health literacy interventions focusing on health care providers, patients, educators, or community organizations in the U.S. Two independent reviewers screened records and extracted data using the Population-Concept-Context framework. Implementation outcomes were assessed using the Reach, Effectiveness, Adoption, Implementation, and Maintenance (RE-AIM) framework to evaluate reporting of Reach, Effectiveness, Adoption, Implementation, and Maintenance dimensions.

**Key Results:**

Of 117 included studies, most targeted health care patients, with strong representation of racial and ethnic groups but limited inclusion of non-English speakers. Health literacy measurement was reported in 71 studies, with only 49 employing validated instruments. The Newest Vital Sign, eHealth Literacy Scale, and RE-AIM were most frequently used. Nearly all studies reported effectiveness with statistically significant results, predominantly measuring behavioral rather than clinical outcomes. RE-AIM analysis revealed substantial reporting gaps, particularly in demographic details, implementation fidelity, cost documentation, and maintenance planning. Only 18% of studies reported fidelity and fewer than 10% described sustainability strategies.

**Discussion:**

While health literacy intervention research has grown substantially, significant inconsistencies in measurement practices and implementation reporting limit comparability and translation into practice. The field requires greater standardization in measurement approaches, more comprehensive demographic reporting to assess equity impacts, and systematic application of implementation science frameworks from intervention inception. Improved reporting standards are essential to accelerate translation of health literacy research into sustainable, equitable practice.

## Abstract (plain language summary)

**Plain Language Summary:**This review examined how health literacy was measured and reported in 117 U.S.-based health literacy intervention studies published between 2014 and 2024. Although many studies included diverse populations, few included non-English speakers. Health literacy was measured in 71 studies, but only 49 used validated tools. Most studies reported whether interventions were effective, but important information about how the interventions were implemented, their costs, and whether they could be sustained over time was often missing. More consistent measurement and reporting are needed to help translate health literacy research into effective, equitable, and sustainable practice.

Health literacy has been defined as the ability to access, understand, and use health information to make informed decisions (World Health Organization [WHO], 2025). It is a foundational component of both individual and population health, influencing how people interpret medical advice, follow treatment plans, engage in preventive behaviors, and navigate health care systems (Coughlin et al., 2020; Kirby & Lindly, 2024). Healthy People 2030 defines the behavioral practice of health literacy not only as a set of individual skills, such as reading, numeracy, and critical thinking, but also as a shared responsibility that includes the obligation of health care organizations to provide clear, equitable, and actionable information (Office of Disease Prevention and Health Promotion, 2020). This dual perspective reflects a shift from viewing health literacy as a patient shortcoming toward recognizing the role of health systems in shaping the accessibility and usability of health information. Building on these definitions, health literacy has been conceptualized as a multidimensional construct. Nutbeam (2000) proposed three progressive levels of health literacy: functional health literacy, which refers to basic reading and numeracy skills necessary to function effectively in health care settings; interactive (or communicative) health literacy, which involves more advanced cognitive and social skills that enable individuals to actively engage with health care providers and apply new information; and critical health literacy, which encompasses the ability to critically analyze information and exert greater control over life events and health-related decisions. This conceptual framework indicates that health literacy extends beyond reading comprehension to include empowerment, shared decision-making, and social participation.

In the United States, nearly 9 in 10 adults experience difficulty understanding and applying everyday health information (National Library of Medicine [NLM], 2024). Low health literacy is associated with poor health outcomes, reduced engagement in preventive services, increased hospitalizations, and higher health care costs (Schillinger et al., 2002; Scott et al., 2002; Shahid et al., 2022; Weiss & Palmer, 2004). Individuals with limited health literacy may encounter difficulties understanding prescriptions, navigating insurance systems, or communicating with providers. These challenges are often exacerbated by structural inequities, such as language barriers, low educational attainment, and fragmented care delivery systems (Al Shamsi et al., 2020; Fleary & Ettienne, 2019; Muvuka et al., 2020). Addressing these disparities requires focused health literacy efforts as well as reliable ways to measure their effectiveness.

Over the past decade, a variety of health literacy interventions have been developed to improve knowledge, communication, and engagement across diverse populations and settings. These include community-based education programs, culturally tailored initiatives, and provider-focused training designed to enhance communication and patient empowerment (Gibson et al., 2022; Islam et al., 2018). Many of these interventions show promise in improving clinical outcomes and promoting health equity, particularly for underserved groups managing chronic conditions (Baur et al., 2018). However, understanding which programs are most effective and how they are adopted, implemented, and sustained requires more than just outcome evaluation. Implementation science offers a framework for studying the real-world impact of interventions, helping to bridge the gap between research and practice (Neta et al., 2018). The Reach, Effectiveness, Adoption, Implementation, and Maintenance (RE-AIM) framework is particularly useful for evaluating the broader impact of health literacy interventions beyond efficacy alone (Glasgow et al., 1999). It allows researchers and practitioners to assess how well programs engage target populations, are integrated into settings, and are maintained over time (Gaglio et al., 2013; Glasgow et al., 2019). RE-AIM was selected for this article because it provides a pragmatic and structured framework for assessing the public health impact of interventions across multiple dimensions that extend beyond efficacy. While other implementation frameworks, such as the Consolidated Framework for Implementation Research, focus primarily on contextual determinants influencing implementation processes, RE-AIM offers quantifiable domains that align with reporting practices commonly described in intervention articles. Given this article's objective to examine how implementation outcomes are reported in published studies, RE-AIM was considered particularly suitable for systematically evaluating reach, effectiveness, adoption, implementation fidelity, and maintenance across diverse intervention types.

A variety of tools have been developed to assess health literacy, including well-established instruments like the Rapid Estimate of Adult Literacy in Medicine, Short Form (REALM) (Arozullah et al., 2007), the Newest Vital Sign (NVS) (Shealy & Threatt, 2016), and the Test of Functional Health Literacy in Adults (TOFHLA) (Parker et al., 1995). Although widely used, legacy performance-based instruments such as REALM and TOFHLA primarily assess functional literacy skills (e.g., reading comprehension and numeracy) and may only partially align with broader, contemporary definitions of health literacy that include interactive and critical dimensions (Berkman et al., 2004; Nutbeam, 2000). Despite advances in conceptualizing health literacy as a multidimensional construct, measurement practices in intervention research continue to rely heavily on tools that emphasize functional skills. These tools provide validated ways to assess individual-level health literacy, particularly in patient education settings. However, in applied practice, many programs rely on adapted, locally developed, or nonvalidated tools. These adaptations may be necessary to address linguistic diversity, cultural relevance, literacy level, or practical constraints such as time and staffing (Dijkman et al., 2023; Rademakers et al., 2020). While such flexibility can improve contextual fit and participant engagement, it also introduces variability in how health literacy is measured across studies. This inconsistency makes it challenging to compare outcomes, synthesize evidence, and identify which tools are most appropriate for specific populations or intervention goals (Kiechle et al., 2015; Sawyers et al., 2022). Given the expanding use of health literacy interventions across diverse settings and populations, it is essential to systematically document how measurement tools (both validated and nonvalidated) are being used in practice.

While previous research has contributed valuable insights into the effectiveness and implementation of health literacy interventions, an updated synthesis is needed to reflect more recent programs and reporting practices. Previous studies have reviewed commonly used health literacy measurement tools and their application in evaluating interventions (Faux-Nightingale et al., 2022; Marshall et al., 2025; Tavousi et al., 2022). However, gaps remain in understanding how these tools are used within U.S.-based interventions, particularly regarding the inclusion of both validated and nonvalidated tools and their application across diverse health topics and settings. A scoping review design was selected because the goal of this paper was to map the breadth and characteristics of measurement tools and reporting practices across a heterogeneous body of intervention research, rather than to evaluate intervention effectiveness or estimate pooled effect sizes. Scoping reviews are particularly appropriate when examining variability in concepts, instruments, and implementation practices, and when the goal is to identify gaps and inform future research directions rather than determine causal impact. In light of these considerations, this scoping review had two aims. The primary aim was to map the measurement tools—both validated and nonvalidated—used to assess health literacy outcomes in U.S.-based interventions published between 2014 and 2024. Specifically, we examined the types of instruments used, their validation status, and their application across populations and health topics. The secondary aim was to evaluate how implementation outcomes of these interventions were reported, using the RE-AIM framework to assess patterns in reach, effectiveness, adoption, implementation, and maintenance reporting. By distinguishing measurement practices from implementation reporting, this article seeks to clarify current patterns in health literacy intervention research and identify methodological and reporting gaps that may limit translation into practice.

## Methods

This scoping review followed the updated methodological framework from the Joanna Briggs Institute (JBI) as outlined by Peters and colleagues (2020) and adhered to the Preferred Reporting Items for Systematic Reviews and Meta-Analyses extension for Scoping Reviews guidelines (Tricco et al., 2018). The protocol was developed a priori and registered in the Open Science Framework with the following registration number: osf.io/uk89s. Database selection and the search strategy were developed in consultation with a library scientist. This search was conducted in Ovid MEDLINE on January 16, 2025, and the complete search strategy is provided in **Figure 1**. The starting year of 2014 was selected to capture contemporary health literacy interventions published following the widespread adoption of updated health literacy frameworks and implementation science approaches, while also reflecting the most recent decade of research activity.

**Figure 1.**
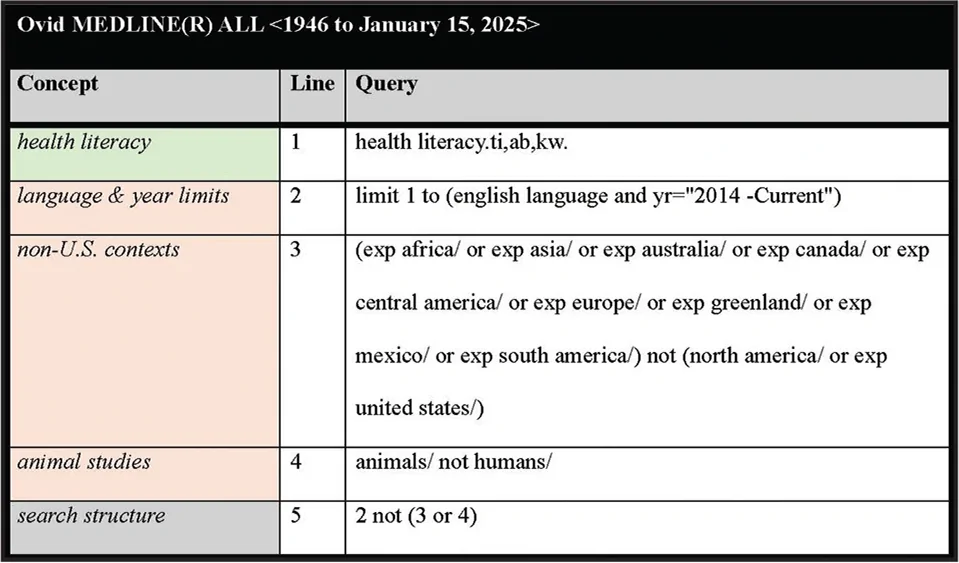
Ovid MEDLINE search strategy used to identify English-language studies on health literacy conducted outside the United States and published through January 15, 2025. The search combined health literacy terms with geographic, language, publication-year, and human-study limits.

### Eligibility Criteria

All study designs reporting primary data on health literacy interventions targeting health care providers, patients, educators, or community advocates in the U.S. were considered eligible. Studies were included if they described any tool (validated or not validated) used to assess health literacy outcomes within these interventions. Eligible articles were published in English between 2014 and 2024. Letters to the editor, commentaries, perspectives, and study protocols were excluded from the primary analysis but were reviewed for contextual insights. No restrictions were applied based on participant age, health setting, or geographic region within the U.S. For the purpose of this article, a “health literacy intervention” was defined as any structured program, strategy, or educational initiative implemented within a health care or community setting that explicitly measured health literacy as an outcome. Interventions were not required to be exclusively designed to improve health literacy; rather, inclusion required that health literacy was assessed as an outcome using a defined instrument as part of the study's evaluation. This operational definition allowed inclusion of broader health-related interventions (e.g., communication training, disease education programs) in which health literacy was measured.

### Study Selection

All identified records were imported into Covidence, a systematic review management tool, for screening and selection. In the first round of review, all titles and abstracts were screened. In the second round of review, full texts were retrieved for studies deemed potentially relevant and screened. Articles were reviewed independently by two reviewers. Discrepancies were resolved through discussion or, if needed, by a third reviewer. Inter-rater reliability between the two reviewers was assessed using the Kappa statistic, yielding a value of 0.78, indicating substantial agreement.

A total of 11,157 studies were imported for screening. After removing 1,051 duplicates, 10,106 records remained for title and abstract screening. Of these, 9,776 were excluded for not meeting eligibility criteria. The remaining 330 articles were retrieved for full-text review. Following full-text assessment, 213 articles were excluded: 4 were not peer-reviewed articles published in English, 1 was published outside of the eligible date range, 128 did not describe health literacy interventions focused on adults, and 79 were conducted outside of the U.S. A total of 117 studies were included in the final review as demonstrated in the PRISMA (preferred reporting items for systematic reviews and meta-analyses) diagram found in **Figure 2**.

**Figure 2.**
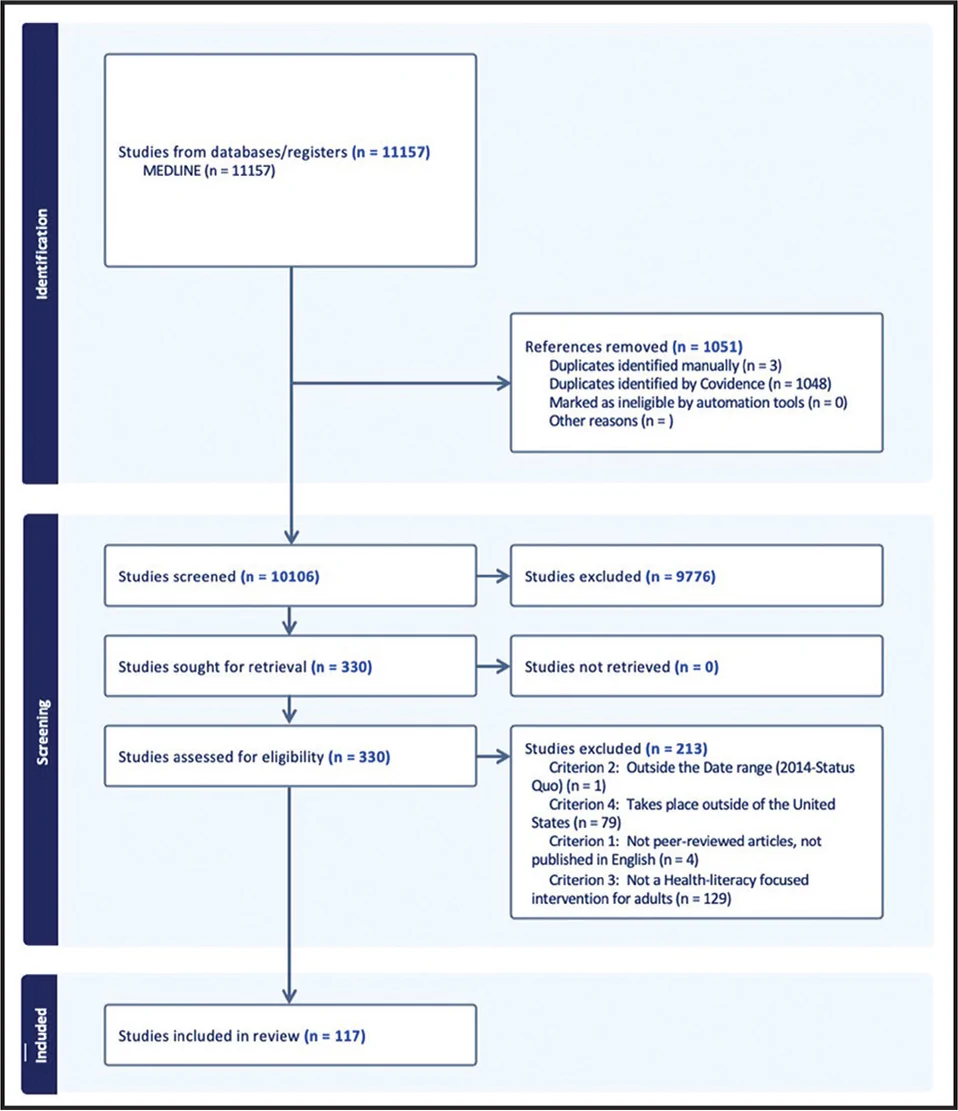
PRISMA (preferred reporting items for systematic reviews and meta-analyses) flow diagram showing the identification, screening, eligibility assessment, exclusion, and final inclusion of studies in the scoping review.

### Data Extraction

Data extraction was guided by the Population–Concept–Context framework. Two reviewers independently extracted data from each eligible study. Extracted information included: bibliographic details (e.g., author names, year of publication), study design, and methodological approach (quantitative, qualitative, or mixed methods). Data were also collected on sample size and population characteristics (e.g., patient, provider, student), including race and ethnicity, gender, and primary language. For each study, specific health literacy topic, geographic scope (national or regional), setting (clinic-based or community-based), and funding source were documented. Health literacy measurement practices were collected, including whether a validated or nonvalidated tool was used and, when applicable, the specific instrument name. A “validated” instrument was defined as a tool with previously published psychometric evaluation demonstrating reliability (e.g., internal consistency, test-retest reliability) and validity (e.g., construct, criterion, or content validity) in peer-reviewed literature.

Similar to previous research (Messiah et al., 2020; Sanchez-Flack et al., 2020; Yudkin et al., 2024), this scoping review assessed each included article's reported outcomes within the RE-AIM implementation science framework (Glasgow et al., 1999) using a modified checklist created by the Implementation Science Team at the National Cancer Institute to score National Institutes of Health (NIH) grant proposals but easily adapted for policy/program analysis and journal articles (NIH, 2019). “Reach” was defined by the geographic setting, sample size, eligibility criteria, assessment of selection bias, and sample characteristics, including child sex, race and ethnicity, socioeconomic status, age, and language spoken. “Effectiveness” was defined by the study design, use of validated measurement tools and primary outcome type. Specifically, effectiveness was conceptualized to assess if effectiveness was measured and if statistical significance was reported. The primary outcome was categorized as to whether it describes a clinical outcome (e.g., HbA1c [hemoglobin A1c] level), behavioral outcome (change in food consumption), or a social determinant of health (e.g., educational level). “Adoption,” a nested, multilevel concept, was defined by participant adoption rate (i.e., referred, consent, enrolled, and retained) and satisfaction, articulation of facilitator and facilitator satisfaction, and the intervention financing source(s). “Implementation” was defined by capturing the use of a theoretical framework, attention to protocol fidelity and adaptations, reporting barriers to implementation, intervention length and/or intensity, measures of cost, measuring the cost of actual delivery, intervention intensity, delivery mode (i.e., whether it was geared towards individuals, groups, or both), and intervention setting. “Maintenance” was defined by the measure of follow-up after the intervention ended, including outcomes measurements, current program status, and funding source.

### Data Synthesis

Extracted data were synthesized using both descriptive statistics and thematic analysis. Frequency tables and visual displays were used to illustrate the distribution of study characteristics, measurement tools, and intervention formats. A thematic analysis was conducted to explore patterns in program content, outcome domains, and gaps in the literature.

## Results

### Population Characteristics

At the study level, most included studies focused on health care patients (*n* = 87, 74.36%), whereas fewer studies targeted health care providers (*n* = 25, 21.37%) or health care students (*n* = 9, 7.69%). Most included both men and women (*n* = 108, 92.31%), while some included only women (*n* = 6, 5.12%) or only men (*n* = 3, 2.56%). Most studies (*n* = 75, 64.10%) focused on communities of color populations, while a smaller portion (*n* = 42, 35.90%), focused on general (*n* = 2, 1.70%) or unspecified racial/ethnic groups (*n* = 40, 34.19%). English-speaking patients were the predominant focus (*n* = 87, 74.36%), though a portion of studies (*n* = 22, 18.80%) included or focused exclusively on Spanish-speaking families. 38 (32.48%) studies focused on populations living with a specific disease, such as type 2 diabetes, HIV, and cancers. Sample sizes varied, with a mean of 202.85 participants across all studies (standard deviation = 305.34, range = 5 to 2011).

### Methodological Characteristics and Effectiveness

Most studies used only quantitative methods (*n* = 106; 90.60%), while 4.27% (*n* = 5) employed only qualitative approaches, and 3.42% (*n* = 4) utilized mixed methods designs. Two studies (1.71%) employed other methods, including a quality improvement approach using the Plan-Do-Study-Act cycle, a structured framework for iterative testing of interventions (Azhar et al., 2022), and a Community-Based Participatory Research project that involved health care providers to codevelop materials with community members (Willis et al., 2020). Regarding study design, 33 (28.21%) were randomized controlled trials, 15 (12.82%) were longitudinal studies (e.g., cohort), and 57 (48.72%) were nonrandomized experimental studies. Geographically, 15.4% were national in scope, and 84.6% were regional. Most interventions (*n* = 61, 52.14%) were conducted in clinic-based settings, while 53 (45.30%) were community-based. Of the 117 included studies, 115 (98.3%) reported their intervention was effective, and 109 (93.16%) reported statistically significant findings. However, only 88 studies (75.21%) explicitly presented their statistically significant results in the article. We did not observe a consistent pattern suggesting that studies using validated instruments were more or less likely to report statistically significant intervention effects compared to those using nonvalidated tools. High rates of reported effectiveness were observed across both groups, which may reflect broader trends in intervention reporting rather than instrument type.

### Measurement of Health Literacy

Health literacy measurement was reported in 71 studies, with 49 used instruments with prior published psychometric validation. The most used instruments included: NVS (*n* = 10) (Castañeda et al., 2015; David et al., 2017; Han et al., 2018, 2019; Sanders et al., 2021; Shiyanbola et al., 2022; Shreffler-Grant et al., 2018, 2021; Son et al., 2024; Tucker et al., 2019); eHealth Literacy Scale (eHEALS) (*n* = 8) (Chenneville et al., 2021; Manganello et al., 2024; Nahm et al., 2019; Nokes & Reyes, 2019; Sanders et al., 2021; Tomsho et al., 2024; Vazquez et al., 2023; Yee et al., 2014); Rapid Estimate of Adult Literacy in Medicine (REALM) (*n* = 7) (Davis et al., 2024; Griffey et al., 2015; Sanders et al., 2021; Wang et al., 2015; Wolf et al., 2014; Yee et al., 2014; Zullig et al., 2014); Chew et al.'s (2004) Health Literacy Screening Tool (*n* = 5) (Castañeda et al., 2015; Hickman et al., 2021; Hillyer et al., 2020; Khanchandani et al., 2022; McNeill et al., 2019). Of the remaining 22 studies, health literacy was assessed using nonvalidated or study-specific tools, most commonly researcher-developed surveys and self-report surveys.

Across the 71 studies that reported health literacy measurement, most instruments assessed functional health literacy, particularly reading comprehension and numeracy skills, consistent with Nutbeam's functional level. Performance-based instruments such as REALM and NVS required participants to read or interpret health-related materials and were administered in clinical or educational settings. In contrast, self-report instruments such as the eHEALS assessed perceived digital health literacy skills and more closely aligned with interactive or communicative dimensions of health literacy. However, few studies explicitly measured critical health literacy, defined as the ability to critically evaluate information and engage in collective or empowerment-oriented decision-making. In terms of scope, most instruments were generic health literacy scales designed for broad health care contexts. A smaller subset of studies employed disease-specific measures tailored to conditions such as diabetes, HIV, or cancer. This distinction is important, as individuals with adequate general literacy skills may experience limited health literacy within specific disease contexts. **Table A** summarizes the validated and nonvalidated health literacy instruments identified across included studies, categorized by mode of assessment, alignment with Nutbeam's conceptual levels, and scope (generic versus disease-specific).

**Table A.** Thematic breakdown of studies included

**Category**	**Operationalized Definition**	**Title of Study**	**Author**	**Date**
**General Health Literacy (n = 29)**	Studies focusing on general health literacy concepts, health literacy assessment, or broad health literacy improvement interventions without focus on specific health conditions.			
		Using Standardized Patient Assessments to Evaluate a Health Literacy Curriculum	Marion et al	2018
		Impacts of educational interventions of librarian instruction on health information seeking attitudes and behaviors in an employee wellness program	Foy	2024
		Effects of an eHealth Intervention on Patient- Provider Interaction and Functional Health Literacy in Adults With Hypertension	Hickman et al	2021
		Evaluating the Effectiveness of the How to Talk to Your Doctor HANDbook Program	McNeill et al	2019
		Improving Health Equity Through Health Literacy Education	Nepps et al	2023
		Enhancing the health knowledge and health literacy of recently resettled refugees through classroom-based instructional methods	Agrawal et al	2024
		The impact of teach-back on comprehension of discharge instructions and satisfaction among emergency patients with limited health literacy: A randomized, controlled study	Griffey et al	2015
		Training Meals on Wheels Volunteers as Health Literacy Coaches for Older Adults	Rubin et al	2014
		Addressing health literacy through clear health communication: A training	Green et al	2014

**Table:** 

		program for internal medicine residents		
		Developing Health Literacy Knowledge and Skills Through Case-Based Learning	Ha & Lopez	2014
		The Impact of a Faculty Development Program in Health Literacy and Ethnogeriatrics	Evans et al	2014
		Parents as Teachers Health Literacy Demonstration Project: Integrating an Empowerment Model of Health Literacy Promotion Into Home-Based Parent Education	Carroll et al	2015
		A Multisite Community-Based Health Literacy Intervention for Spanish Speakers	Soto Mas et al	2015
		The Health Literacy and ESL Study: A Community-Based Intervention for Spanish- Speaking Adults	Soto Mas et al	2015
		Long-Term Effects of a Health Literacy Curriculum for Medical Students	Coleman et al	2016
		Long-term Effects of a Health Literacy Curriculum for Family Medicine Residents	Coleman et al	2017
		Perception Versus Reality: The Use of Teach Back by Medical Residents	Feinberg et al	2019
		A literacy-sensitive approach to improving antibiotic understanding in a community-based setting	David et al	2017
		An Interactive, Multifaceted Approach to Enhancing Pharmacy Students' Health Literacy Knowledge and Confidence	Mnatzaganian et al	2017
		The Teach Back Project: A System-wide Evidence Based Practice Implementation	Klingbeil & Gibson	2018

**Table:** 

		Enhancing organizational health literacy in a rural Missouri clinic: a qualitative case study	Wray et al	2019
		Health Literacy Initiatives and Lessons Learned with NGOs: Wisconsin Health Literacy	LaScala et al	2020
		Evaluating a Health Literacy Communication Training for Medical Students: Using Plain Language	Hildenbrand et al	2020
		Teach-Back Training for Nursing Students: Knowledge and Beliefs After Training for Health Literate Communication	Feinberg et al	2021
		You Can Teach Every Patient: A Health Literacy and Clear Communication Curriculum for Pediatric Clerkship Students	Spengler et al	2021
		Cost-Health Literacy as an Educational Objective in Fellowship Training	Fischer et al	2022
		Improving Health Literacy Knowledge, Behaviors, and Confidence with Interactive Training	Gibson et al	2022
		Elements for successful implementation of a clinic-based health literacy intervention	Macauda et al	2022
		Promoting Health Literacy Among Adult ELLs Virtually During COVID-19	Shashikiran et al	2023
**Cancer (n = 12)**	Studies focusing on various cancer types, cancer screening, prevention, treatment education, and cancer-related health literacy across different organ systems.			
		The impact of curated educational videos on pathology health literacy for patients with a pancreatic, colorectal, or prostate cancer diagnosis	Khanchandani et al	2022

**Table:** 

		Assessing the impact of the skin cancer awareness now (SCAN!) intervention several months following the intervention	Dhumal et al	2023
		Testing a Breast Cancer Screening Decision aid Designed for Health Literacy Accessibility	Gunn et al	2024
		Improving Patient Understanding and Outcomes in Lung Cancer Using an Animated Patient's Guide with Visual Formats of Learning	Patel et al	2025
		Impact of a literacy-sensitive intervention on CRC screening knowledge, attitudes, and intention to screen	Hodgens et al	2016
		Video-Based Educational Tool Improves Patient Comprehension of Common Prostate Health Terminology	Wang et al	2015
		Academic-Community Partnership to Develop a Patient-Centered Breast Cancer Risk Reduction Program for Latina Primary Care Patients	Castañeda et al	2015
		Breast and Cervical Cancer Screening Literacy Among Korean American Women: A Community Health Worker-Led Intervention	Han et al	2017
		Improving Cancer Patients' Insurance Choices (I Can PIC): A Randomized Trial of a Personalized Health Insurance Decision Aid	Politi et al	2020
		Community education to enhance the more equitable use of precision medicine in Northern Manhattan	Hillyer et al	2020
		Preliminary Evaluation of a Citizen Scientist Educational	Matthews et al	2022

**Table:** 

		Curriculum Aimed at Engaging Black Men in Lung Cancer Early Detection Screening		
		An accessible, relational, inclusive, and actionable (ARIA) model of genetic counseling compared with usual care: Results of a randomized controlled trial	Joseph et al	2022
**Mental Health (n = 9)**	Studies focusing on mental health conditions, psychological wellbeing, stress management, and mental health literacy including depression, anxiety, trauma, and stigma around mental health conditions.			
		Research Brief Improving Mental Health Literacy in Nursing Students: Integrating Mental Health Throughout the Curriculum	Gamez & Gunning	2024
		Mental health priorities and cultural-responsiveness of the Mental Health First Aid (MHFA) training for Asian immigrant populations in Greater Boston, Massachusetts	Kim et al	2024
		Growing Resilience in Tough Times (GRITT): Development and Randomized Trial of a Farmer Mental Health Literacy Intervention	Holmstrom & Shelle	2024
		A Pilot Randomized Controlled Trial of the Family Assessment and Feedback Intervention (FAFI): Effects on Mental Health Literacy and Attitudinal Engagement with Health Supports and Services	Ivanona et al	2026

**Table:** 

		Improving Mental Health Literacy and Stigma Among the Hmong	Vang-Kue et al	2025
		Evaluation of Mental Health First Aid USA Using the Mental Health Beliefs and Literacy Scale	Banh et al	2019

		Culturally Adapting Youth Mental Health First Aid Training for Asian Americans	Wang et al	2022
		¡Yo no Estoy Loca! A Behavioral Health Telenovela Style Entertainment Education Video: Increasing Mental Health Literacy Among Latinas	Gonzalez & Benuto	2022
		An Effective Mental Health Literacy Program for Farm Financial Service Providers	Cuthberston et al	2023
**HIV / AIDS (n = 5)**	Studies related to HIV/AIDS education, prevention, treatment adherence, and related health literacy including PrEP, PEP, and opportunistic infections.			
		Wisdom Matters: Honoring the Wisdom and Assessing the Health Literacy of Black Women Living with HIV	Amutah-Onukagha et al	2021
		Acceptability and Feasibility of “Latinos Unidos”: A Microgame Resource Combatting Health Misinformation for Latinos Living with HIV	Dunleavy et al	2024
		Efficacy of an HIV/AIDS and HIV Testing Video for Spanish-Speaking Latinos in Healthcare and Non-Healthcare Settings	Merchant et al	2015
		Can a brief peer-led group training intervention improve health literacy in persons living with HIV?	Sanders et al	2021

**Table:** 

		Results from a randomized controlled trial		
		Bijou: Engaging Young MSM in HIV Care Using a Mobile Health Strategy	Chenneville et al	2021
**Maternal / Women's Health (n = 4)**	Studies addressing women's reproductive health, maternal health, menstrual health, prenatal care, and women-specific health conditions and literacy needs.			
		Participatory group prenatal education using photonovels: Evaluation of a lay health educator model with low-income Latinas	Auger et al	2015
		Efficacy of the Flo App in Improving Health Literacy, Menstrual and General Health, and Well-Being in Women: Pilot Randomized Controlled Trial	Cunningham et al	2024
		A randomized trial of a prenatal genetic testing interactive computerized information aid	Yee et al	2014
		The Use of a Community- Based Preconception Peer Health Educator Training Intervention to Improve Preconception Health Knowledge	King et al	2019
**Oral Health (n = 3)**	Studies addressing oral health literacy, dental care, and dental health education.			
		Evaluating augmented reality e-typodont to improve a patient's dental implant health literacy	Sukotjo et al	2025
		Improving parent oral health literacy in Head Start programs	Dudovitz et al	2020
		Microbiology-Based Instruction during Prenatal Dental Visits Improves Perinatal Oral Health Literacy	Thomson et al	2022

**Table:** 

**Aging / Geriatric Care (n = 2)**	Studies focusing on health literacy needs of older adults, geriatric-specific health conditions, and aging-related health concerns.			
		Individualistic Versus Collaborative Learning in an eHealth Literacy Intervention for Older Adults: Quasi-Experimental Study	Vazquez et al	2023
		Theory-driven role model stories improve palliative care knowledge among a diverse older population	Hoe et al	2021
**Complementary & Alternative Medicine (n = 2)**	Studies focusing on complementary and alternative medicine (CAM) health literacy, integrative health approaches, and non-conventional treatments.			
		Bee SAFE, a Skill-Building Intervention to Enhance CAM Health Literacy: Lessons Learned	Shreffler-Grant et al	2018
		Community-based Skill Building Intervention to Enhance Health Literacy Among Older Rural Adults	Shreffler-Grant et al	2021
**Chronic Disease Management (n = 15)**	Studies addressing health literacy related to long-term health conditions requiring ongoing medical care and self-management, including diabetes, cardiovascular disease, obesity, hypertension, and multiple chronic conditions. Focus on disease prevention, treatment adherence, lifestyle modifications, and patient education for chronic illness management.			
Diabetes Studies				
		Mobile Virtual Reality Versus Mobile 360° Video to Promote Enrollment in the Diabetes Prevention Program	Gibson et al	2022

**Table:** 

		Among Hispanic Adults: Pilot Study		
		Feasibility of a Randomized Controlled Mixed Methods Trial to Address Health Literacy, Beliefs, Medication Adherence, and Self-Efficacy (ADHERE) in a Clinical Pharmacist-Led Clinic	Shiyanbola et al	2022
		Implementation and Evaluation of a Low Health Literacy and Culturally Sensitive Diabetes Education Program	Swavely et al	2014
		Improving Medication Understanding Among Latinos Through Illustrated Medication Lists	Mohan et al	2014
		Improving Diabetes Health Literacy by Animation	Calderón et al	2014
		Text Messaging May Engage and Benefit Adults with Type 2 Diabetes Regardless of Health Literacy Status	Bergner et al	2017
		Health literacy enhanced intervention for inner-city African Americans with uncontrolled diabetes: a pilot study	Han et al	2018
		Effectiveness of DVD vs. group-initiated diabetes prevention on information uptake for high & low health literacy participants	Goessl et al	2019
Cardiovascular Studies				
		A Health Literacy-Focused Intervention for Latinos with Hypertension	Han et al	2018
		Developing, optimizing, and evaluating patient infographics for diagnosing cardiac amyloidosis	Pack et al	2023
		A Culturally Appropriate Educational Intervention Can Improve Self-Care in Hispanic Patients With Heart Failure: A Pilot Randomized Controlled Trial	Howie-Esquivel et al	2014

**Table:** 

		Fostering African-American Improvement in Total Health (FAITH!): An Application of the American Heart Association's Life's Simple 7^TM^ among Midwestern African-Americans	Brewer et al	2017
Obesity Studies				
		The Stress of Advancement: A Nurse Practitioner's Exploration in Providing Culturally Competent Obesity Prevention Counseling in Black Women	Berry et al	2024
		Health Literacy Directed Weight Loss Intervention in Primary Care Clinics	Davis et al	2024
General Chronic Disease				
		The Effects of a Theory-Based Patient Portal e-Learning Program for Older Adults with Chronic Illnesses	Nahm et al	2019
**Healthcare Delivery & Communication (n = 12)**	Studies focusing on improving health literacy through enhanced patient-provider communication, healthcare professional training, cultural competency, and healthcare system improvements. Includes communication skills training, teach-back methods, clear health communication, intercultural competence, and quality improvement initiatives in healthcare delivery.			
Health Communication Studies				
		Improving Patient Health Literacy During Telehealth Visits Through Remote Teach-Back Methods Training for Family Medicine Residents: Pilot 2-Arm Cluster, Nonrandomized Controlled Trial	Barksdale et al	2023

**Table:** 

		Teaching and Learning Health Literacy in a Doctor of Pharmacy Program	Trujillo & Figler	2015
		A Health Literacy Training Intervention for Physicians and Other Health Professionals	Coleman & Fromer	2015
		A Nurse Leadership Project to Improve Health Literacy on a Maternal-Infant Unit	Stikes et al	2015
		Development and Implementation of the How to Talk to Your Doctor HANDbook Health Literacy Program in Rural Counties	Washburn et al	2019
		Health Literacy and Clear Bedside Communication: A Curricular Intervention for Internal Medicine Physicians and Medicine Nurses	Allenbaugh et al	2019
		A Brief Communication Curriculum Improves Resident and Nurse Communication Skills and Patient Satisfaction	Allenbaugh et al	2019
Health Literacy Training Studies				
		Evaluating the Effectiveness of Using the Teach-Back Method to Improve the Health Literacy of Individuals in the Community	Brown et al	2025
		Development, implementation, and evaluation of Teach Back curriculum for community health workers	Holcomb et al	2022
Intercultural Competency Studies				
		A Novel Approach to Improve Health Literacy in Immigrant Communities	Johnson et al	2019
		Health Literacy and Intercultural Competence Training	Mavreles Ogrodnick et al	2021
Quality Improvement Studies				
		Demonstration of the Health Literacy Universal	Mabachi et al	2016

**Table:** 

		Precautions Toolkit: Lessons for Quality Improvement		
**Musculoskeletal & Neurological Health (n = 6)**	Studies focusing on health literacy for conditions affecting the musculoskeletal system (bones, joints, muscles), nervous system (brain, spinal cord, nerves), and sensory organs. Includes orthopedic conditions, neurological disorders, cognitive health, movement disorders, vision care, and related patient education interventions.			
Musculoskeletal Studies				
		A Video-Based Intervention for Increasing Health Literacy in Total Joint Arthroplasty Patients at a Safety-Net Hospital: A Prospective Single-Blind Cohort Study	Son et al	2024
		Efficacy study of multimedia rheumatoid arthritis patient education program	Unk & Brasington	2014
		Comparison of multimedia and printed patient education tools for patients with osteoporosis: a 6-month randomized controlled trial	Lopez-Olivo et al	2020
		Health Literacy in an Orthopaedic Trauma Patient Population: Improving Patient Comprehension with Informational Intervention	Tsahakis et al	2014
Neurological Studies				
		A boot camp translation of Alzheimer's disease in Hispanic/Latino communities	Medina et al	2023
		Use of Three-Dimensional Printed Brain Models During Deep Brain Stimulation Surgery Consultation for Patient Health Literacy: A Randomized Controlled Investigation	Hirt et al	2022
		Quality Improvement in Delirium Health Literacy in	Azhar et al	2022

**Table:** 

		Older Adult Patients and Their Caregivers Attending a Geriatric Clinic		
Eye Health Studies				
		African American patient-provider communication about glaucoma vision quality-of-life	Sleath et al	2024
**Technology & Tools (n = 5)**	Studies examining health literacy interventions using digital technologies, specialized medical procedures education, and tools for improving health information comprehension. Includes electronic health literacy, mobile health applications, medical documentation simplification, and technology-enhanced patient education for complex medical procedures.			
Digital / Technology Health Literacy Studies				
		Get Health'e': A Pilot Test of a Digital Health Literacy Intervention for Young Adults	Manganello et al	2024
		Do Brief Educational Sessions Increase Electronic Health Literacy of Low-Income Persons Living With HIV/AIDS?	Nokes & Reyes	2019
Specialized Medical Procedures				
		A printed information leaflet about MRI and radiologists improves neuroradiology patient health literacy	Ginat & Christoforidis	2018
		Evaluating augmented reality e-typodont to improve a patient's dental implant health literacy	Sukotjo et al	2025
Documentation				
		A Smart Mobile App to Simplify Medical Documents and Improve Health Literacy: System Design and Feasibility Validation	Hendawi et al	2022
**Medication & Treatment (n = 3)**	Studies addressing health literacy related to medication

**Table:** 

	understanding, adherence, safe medication use practices, and comprehension of treatment instructions. Focus on medication literacy, prescription comprehension, drug safety education, and interventions to improve medication-related health outcomes.			
		Patient-perceived understanding of home-going medication with transitions of care services at a pediatric institution	Sebastian et al	2024
		A health literacy pilot intervention to improve medication adherence using Meducation® technology	Zullig et al	2014
		Comparative Effectiveness of Patient-centered Strategies to Improve FDA Medication Guides	Wolf et al	2014
**Preventative Care & Health Promotion (n = 7)**	Studies addressing health literacy for disease prevention, health promotion behaviors, and population health initiatives. Includes vaccination education, infectious disease prevention, environmental health awareness, nutritional literacy, and community-based health promotion interventions aimed at preventing illness and promoting wellness.			
Immunization / Vaccines				
		Visually enhanced education and immunization perceptions in low-income parents	Papapchrisanthou & Loman	2018
		Improving Immunization and Health Literacy Through a Community-Based Approach	Willis et al	2020
Infectious Disease Prevention				
		Infectious Disease Education in a Carceral Setting: Insights	Loyd et al	2024

**Table:** 

		from a Jail-Based Medical Student-Led Health Literacy Initiative		
		Addressing COVID-19 and Health Literacy Disparities Among Correctional Facility Residents Through Dialogue-Based Education	Kader et al	2024
Environmental Health				
		Improving the Health and Environmental Health Literacy of Professionals: Evaluating the Effect of a Virtual Intervention on Phthalate Environmental Health Literacy	Tomsho et al	2024
		Nutrition and Environmental Pollution Extension Curriculum Improved Diet-Related Behaviors and Environmental Health Literacy	Brewer et al	2019
		Increasing Environmental Health Literacy through Contextual Learning in Communities at Risk	Davis et al	2018
Nutrition				
		A Culturally Sensitive Church-Based Health-Smart Intervention for Increasing Health Literacy and Health-Promoting Behaviors among Black Adult Churchgoers	Tucker et al	2019

### Content Characteristics

The following section describes the thematic focus of intervention content rather than the measurement instruments used to assess health literacy. Among the 117 reviewed articles, the most commonly addressed health literacy topic was general health literacy (*n* = 29, 24.79%), defined as the ability to access, understand, and use health information to make informed decisions (WHO, 2025) and the capacity of providers to deliver clear, equitable, and actionable information to patients (Office of Disease Prevention and Health Promotion, 2020). This category included studies on health literacy frameworks, assessment strategies, and broad interventions aimed at improving health literacy, such as medical student communication training, Teach-Back programs, and community-based literacy interventions. The second most common was chronic disease management (*n* = 15, 12.82%), which encompassed patient health literacy as it relates to the prevention, treatment adherence, and self-management of conditions such as diabetes, cardiovascular disease, and obesity. This was followed by health care delivery and provider communication (*n* = 12, 10.26%), which included interventions to improve patient–provider communication and cultural competence. In several studies, particularly those focused on patient–provider communication, the content of the intervention overlapped conceptually with the domains assessed by health literacy instruments. For example, communication training programs frequently measured self-reported confidence in understanding medical information, potentially capturing related but distinct constructs such as self-efficacy or communication competence. This overlap highlights the importance of clearly distinguishing intervention targets from measurement constructs when interpreting outcomes. Finally, there were several health-specific topics, including cancer (*n* = 12, 10.26%), which addressed cancer screening literacy, treatment education, and survivorship care; mental health (*n* = 9, 7.69%), focusing on mental health literacy, stigma reduction, and culturally adapted interventions; HIV/AIDS (*n* = 5, 4.27%), addressing education and prevention strategies; maternal/women's health (*n* = 4, 3.42%), focusing on prenatal care, menstrual health, and reproductive health literacy; and smaller categories such as oral health, aging/geriatric care, and preventive care & health promotion. A thematic breakdown is provided in **Table 1**.

**Table 1:** Health Literacy Instruments Identified in Included Studies (2014–2024)

**Instrument**	**Validation Status**	**Mode of Assessment**	**HL Level**	**Generic Versus Disease-Specific**	**Number of Studies**
Newest Vital Sign	Validated	Performance-based	Functional	Generic	10
eHealth Literacy Scale	Validated	Performance-based	Functional	Generic	8
Rapid Estimate of Adult Literacy in Medicine	Validated	Performance-based	Functional	Generic	7
Health Literacy Screening Questions	Validated	Performance-based	Functional	Generic	5
Short Test of Functional Health Literacy in Adults	Validated	Performance-based	Functional	Generic	3
MSU CAM Health Literacy Scale	Validated	Self-report	Functional	Generic	2
Mental Health Literacy Scale	Validated	Self-report	Functional + interactive	Disease-specific	2
Brief Health Literacy Screen	Validated	Self-report	Functional	Generic	1
European Health Literacy Survey	Validated	Self-report	Functional + interactive + critical	Generic	1
Health Literacy Instrument for Adults	Validated	Self-report	Functional + interactive	Generic	1
Health Literacy Skills Instrument	Validated	Performance-based	Functional + interactive	Generic	1
Diabetes Health Literacy Survey	Validated	Self-report	Functional	Disease-specific	1
Literacy Assessment in Diabetes	Validated	Performance-based	Functional	Disease-specific	1
Spoken Knowledge in Low Literacy in Diabetes	Validated	Performance-based	Functional	Disease-specific	1
Cancer Health Literacy Test	Validated	Performance-based	Functional	Disease-specific	1
Rapid Estimate of Adult Literacy in Genetics	Validated	Performance-based	Functional	Disease-specific	1
Short Assessment of Health Literacy for Spanish Adults	Validated	Performance-based	Functional	Disease-specific	1
Depression Literacy Questionnaire	Validated	Self-report	Functional	Disease-specific	1
Mental Health Beliefs and Literacy Scale	Validated	Self-report	Functional	Disease-specific	1

**Table:** 

**Instrument**	**Validation Status**	**Mode of Assessment**	**HL Level**	**Generic Versus Disease-Specific**	**Number of Studies**
HBP Health Literacy Scale	Validated	Self-report	Functional	Disease-specific	1
All Aspects of Health Literacy Scale	Validated	Self-report	Functional + interactive + critical	Generic	1
Author-developed HL questionnaires	Not validated	Self-report	Mostly functional	Often disease-specific	22

Note. CAM = complementary and alternative medicine; HBP = high blood pressure; HL = health literacy; MSU = Montana State University.

### Implementation Reporting–RE-AIM

To assess the extent to which implementation outcomes were documented across studies, each article was evaluated using the RE-AIM framework, with results summarized across all five dimensions in **Table B**. The following sections present reporting frequencies for each RE-AIM dimension, highlighting areas of strength and identifying gaps in current implementation reporting practices.

**Table B:** Proportion of health literacy studies that took place in the United States reporting Reach, Effectiveness, Adoption, Implementation, and Maintenance (RE-AIM) indicators and components.

**RE-AIM Dimensions and Components**	**Number Reporting**	**Percentage Reporting**

**Reach**		*Average overall proportion:*

Geographic Setting	109	93.16%

Sample Size	112	95.73%

Eligibility Criteria	105	89.74%

Participant Selection Bias Assessed	24	20.05%

Participant Sex	4	3.41%

Participant Race/Ethnicity	76	64.96%

Participant Age	35	29.91%

Participant Language Spoken	112	95.73%

**Effectiveness**		*Average * *overall * *proportion* *: *

Study Design	116	99.15%

Intervention reported to be effective	“Yes”: 109	93.16%
	Reported in general: 115	98.29%

Statistically Significant Results	“Yes”: 88	75.21%
	Reported in general: 115	98.29%

Measurement tool is validated	256	256/433 = 59.12%

Primary outcome is clinical	1	0.85%

Primary outcome behavioral	115	98.29%

Primary outcome is a social determinant of health (e.g. education level)	0	0.00%

**Adoption**		*Average * *overall * *proportion* *: *

(Adoption) Rate(s)	25	21.37%
Referred	69	58.97%
Consented	81	69.23%
Enrolled	105	89.74%
Retained		

Facilitator is Articulated (Description of staff and level of expertise or training provided)	85	72.65%

Facilitator Satisfaction (Characteristics of adoption/non-adoption)	8	6.84%

Participant Satisfaction (Characteristics of adoption/non-adoption)	44	37.61%

Intervention Financing Reported	86	73.50%

Remote Aspect via eHealth Reported (as eHealth can be either the facilitator and/or delivery mode)	41	35.04%

**Implementation**		*Average overall proportion:*

**Table:** 

Based in a Theoretical Framework	46	39.32%

Fidelity Reported (Extent protocol delivered as intended)	21	17.95%

Barriers Reported	72	61.54%

Intervention Intensity		
Length of time between first and last engagement	90	76.92%
Total number of engagements	106	90.60%
Total amount of time participants were engaged through the entire intervention	76	64.96%

Measures of cost (delivery)	19	16.24%

Intervention Intensity	114	97.44%

	Reported: 117	100.00%
Delivery Mode (i.e., if group or individual or both)	Group: 54	46.15%
	Individual: 45	38.46%
	Both: 18	15.38%

Intervention Setting	112	95.73%

**Maintenance**		*Average overall proportion:*

Follow-up Duration	92	78.63%

Current Status of the Intervention	59	50.42%

Future Funding (Cost of Maintenance)	11	9.40%

Reach. Most studies reported sample size (*n* = 112, 95.7%), language spoken (*n* = 112, 95.7%), and geographic setting (*n* = 109, 93.2%). Eligibility criteria were documented in 105 studies (89.7%). However, demographic reporting was limited: race and ethnicity (*n* = 76, 65%), age (*n* = 35, 29.9%), and sex (*n* = 4, 3.4%). Selection bias was assessed in 24 studies (20.1%).

Effectiveness. Nearly all studies described study design (*n* = 116, 99.2%) and reported intervention effectiveness (*n* = 115, 98.3%). Among these, 109 studies (93.2%) explicitly described interventions as effective, with 88 (75.2%) reporting statistically significant results. Validated measurement tools were used in 256 of 433 outcomes (59.1%). Behavioral outcomes dominated (*n* = 115, 98.3%), while only one study reported a clinical outcome (0.9%), and none reported a social determinant of health as the primary outcome.

Adoption. Participant retention was the most reported (*n* = 105, 89.7%), followed by enrollment (*n* = 81, 69.2%) and consent rates (*n* = 69, 59%), while referral rates were rarely documented (*n* = 25, 21.4%). Facilitator characteristics were described in 85 studies (72.7%), but facilitator satisfaction was less mentioned (*n* = 8, 6.8%). Participant satisfaction was included in 44 studies (37.6%). Intervention financing was reported in 86 studies (73.5%).

Implementation. Most studies reported on intervention intensity, including number of engagements (*n* = 106, 90.6%), length (*n* = 90, 76.9%), and total engagement time (*n* = 76, 65%); overall, 114 studies (97.4%) addressed some aspect of intensity. Delivery mode was universally reported across all studies, with 54 (46.2%) delivered in groups, 45 (38.5%) individually, and 18 (15.4%) using both. Intervention setting was described in 112 studies (95.7%). Theoretical frameworks were cited in 46 studies (39.3%). Barriers to implementation were discussed in 72 studies (61.5%), while fidelity was reported in 21 (18%) and delivery cost in 19 (16.2%).

Maintenance. Maintenance was the least frequently reported RE-AIM dimension. While 78.6% (*n* = 92) of studies documented some follow-up duration, 59 (50.4%) reported on the intervention's current status at the time of publication, and 11 (9.4%) described future funding to sustain the program.

## Discussion

This scoping review aimed to map how health literacy measurement tools (both validated and non-validated) have been used in interventions across the U.S. over the past decade. In addition to identifying measurement tools, a secondary objective of this article was to examine how implementation outcomes were reported across studies using the RE-AIM framework. In synthesizing data from 117 studies published between 2014 and 2024, we found while most interventions reported positive outcomes, the tools used to assess health literacy varied considerably, with only 49 of 71 studies employing validated instruments. Despite the methodological diversity, nearly all studies reported effectiveness, with over 93% citing statistically significant results. These findings underscore both the growing interest in health literacy as a lever for improving health outcomes and the pressing need for greater standardization in measurement practices to enhance comparability and rigor across studies.

Methodologically, the review uncovered substantial variation in how health literacy was measured. Of the 71 studies that reported using a measurement tool, only 49 (69%) employed validated instruments. This pattern may reflect the evolving nature of health literacy intervention research, where investigators often adapt or develop context-specific tools to capture intervention outcomes that are not fully addressed by existing standardized instruments. While these tools are widely accepted, they primarily assess functional health literacy and may not capture broader dimensions such as communicative or critical literacy (Nutbeam, 2000). Previous research has reported low to moderate correlations among commonly used health literacy instruments, suggesting that these tools capture related but distinct constructs of health literacy. For example, studies comparing instruments such as the NVS and REALM have found only moderate associations between measures, reflecting differences in the specific domains assessed, including numeracy, reading comprehension, and broader multidimensional aspects of health literacy (Jessup et al., 2024; Kordovski et al., 2017). In addition, while functional health literacy remains the most commonly measured dimension, fewer included studies explicitly assessed communicative or critical health literacy in this article. One potential explanation is that critical health literacy remains conceptually complex and inconsistently defined across the literature, which has limited the development and adoption of standardized measurement tools (Abel & Benkert, 2022). As the field continues to evolve, researchers and practitioners need to establish consensus on core measurement standards that balance rigor with cultural and contextual flexibility.

Reflecting this measurement heterogeneity, nearly 40% of studies used nonvalidated or locally adapted tools, often to accommodate cultural, linguistic, or contextual needs. Although such adaptations can enhance relevance and engagement, they also introduce challenges related to construct validity, comparability, and reproducibility. This heterogeneity complicates efforts to synthesize findings across studies and limits the ability to identify best practices. The continued use of nonvalidated instruments carries practical consequences that extend beyond individual studies. When instruments lack published psychometric evaluation, it is difficult to determine whether observed changes reflect true shifts in health literacy or are artifacts of measurement error. Reporting gaps associated with nonvalidated tool use, including absent reliability statistics, undefined construct boundaries, and inconsistent administration procedures, make it nearly impossible for journal editors and peer reviewers to evaluate the quality of study findings. For authors, the absence of clear measurement standards may perpetuate a cycle in which locally developed tools are used out of convenience and then reported without sufficient psychometric detail for others to assess or replicate. These weaknesses are not unique to health literacy research and likely reflect broader challenges in intervention science, including inadequate implementation reporting, insufficient demographic detail, and limited attention to fidelity. Greater standardization in how health literacy is measured and reported would benefit the field considerably, and journals could play an important role by requiring authors to report the psychometric properties of any instrument used, whether validated or not.

The measurement patterns identified in this review also reflect a broader conceptual tension in the field. As described in the Introduction, Nutbeam (2000) proposed a progressive model of health literacy moving from functional skills, such as reading and numeracy, toward interactive and critical capacities that enable individuals to engage with, evaluate, and act on health information. Yet the instruments most commonly used in the included studies, including the NVS, REALM, and eHEALS, predominantly capture functional or, in the case of eHEALS, interactive dimensions of health literacy. The near absence of tools measuring critical health literacy suggests that despite two decades of conceptual advancement, measurement practice has not kept pace with theory. This gap has real implications: interventions designed to build empowerment, shared decision-making, and collective action cannot be adequately evaluated using tools designed to assess reading comprehension or numeracy. As the field continues to adopt broader definitions of health literacy, measurement frameworks will need to evolve accordingly.

Aligned with prior reviews (Haun et al., 2014; Sørensen et al., 2012), this scoping review reinforces the foundational role of health literacy in improving health outcomes and reducing disparities. Our synthesis reveals the field is both active and diverse, with interventions spanning clinical care environments, community organizations, academic or training settings, and digital platforms. To maximize the impact of these diverse interventions, the design and evaluation of future health literacy interventions should systematically incorporate organizational health literacy frameworks addressing institutional capacity, communication systems, and structural supports for accessible health information delivery. Most studies focused on patients (74.4%), with a strong emphasis on people of color populations (64.1%) and English-speaking participants (74.4%). While this focus reflects national public health priorities to address inequities in access and outcomes (Office of Disease Prevention and Health Promotion, 2020), it also highlights a potential gap in reaching linguistically diverse populations, particularly those with limited English proficiency. Only 18.8% of studies included or focused on Spanish-speaking participants, despite Spanish being the second most spoken language in the U.S. (U.S. Census Bureau, 2023). This underrepresentation raises concerns about the inclusivity and generalizability of current health literacy interventions.

The reviewed interventions addressed a wide range of health topics and literacy domains, reflecting the field's multidimensional scope and responsiveness to diverse public health needs. These interventions often aimed to improve patient understanding of disease mechanisms, treatment adherence, and self-management, aligning with evidence that condition-specific literacy is linked to improved clinical outcomes (Berkman et al., 2011). Mental health literacy, in particular, was frequently addressed in underserved populations, with interventions incorporating stigma reduction and help-seeking behaviors—key components of mental health promotion (Jorm, 2012). In addition to disease-specific content, many studies focused on functional health literacy, including the ability to navigate health care systems, understand medication instructions, and communicate effectively with providers (Nutbeam, 2000). A smaller but growing subset of studies addressed digital and media health literacy, reflecting the increasing reliance on online health information and telehealth platforms (Norman & Skinner, 2006). However, few interventions explicitly focused on critical health literacy, the capacity to critically evaluate health information and engage in collective action and shared decision making, highlighting a persistent gap identified in this article and an opportunity for future research to adopt more empowerment-oriented approaches (Nutbeam, 2008; Sørensen et al., 2012).

The review also revealed a wide range of settings in which health literacy interventions are being implemented. This distribution highlights the recognition that health literacy is shaped not only by clinical encounters but also by the broader social and environmental contexts in which individuals live and make health decisions (Nutbeam, 2008; Sentell et al., 2020). Despite this diversity, few studies explicitly examined how setting influenced intervention outcomes, and even fewer provided detailed descriptions of implementation processes or contextual adaptations. This gap presents an opportunity for future research to explore how setting-specific factors—such as organizational culture, staffing, or community trust—affect the feasibility, fidelity, and effectiveness of health literacy interventions. Few studies address the use and effectiveness of organizational health literacy measurement tools, especially in the community setting (Stone et al., 2025).

### Implementation Outcomes

The implementation reporting patterns revealed through the RE-AIM analysis illuminate a critical disconnect between what is measured and what is needed for effective translation of health literacy interventions into practice. The strong reporting of basic reach indicators alongside weak documentation of demographic details and selection bias reflects a broader trend in intervention research where surface-level descriptive statistics are prioritized over the deeper contextual information required to assess equity and generalizability (Kessler et al., 2012). This imbalance has meaningful consequences for health literacy research, which explicitly aims to reduce disparities yet cannot adequately assess differential impacts across age, sex, or socioeconomic subgroups without comprehensive demographic reporting. Similarly, the near-universal focus on behavioral rather than clinical outcomes, while practical given typical study timeframes, perpetuates a significant evidence gap identified in previous systematic reviews regarding whether health literacy gains translate into improved health status and reduced health care utilization (Berkman et al., 2011; Sørensen et al., 2012). This pattern suggests that the field may be systematically undercounting its true impact by measuring proximal changes in knowledge or behavior rather than the ultimate health outcomes that justify investment in these interventions. The implications extend beyond academic interest to practical decision-making, as policymakers and health care administrators require evidence of clinical and cost outcomes to support adoption and reimbursement decisions (Glasgow et al., 1999).

The underreporting of adoption, implementation fidelity, cost, and maintenance dimensions represents perhaps the most consequential finding from this RE-AIM analysis, as these are precisely the factors that determine whether evidence-based interventions can be successfully scaled and sustained beyond research settings. The sparse documentation of implementation fidelity aligns with previous implementation science research showing that fewer than twenty percent of intervention studies adequately report what was actually delivered versus what was intended, making replication nearly impossible (Carroll et al., 2007; Wiltsey Stirman et al., 2012). Without fidelity data, practitioners cannot determine which intervention components are essential versus adaptable, leading to either rigid implementation that fails to fit local contexts or overly flexible adaptation that undermines effectiveness (Proctor et al., 2011). The minimal attention to cost reporting is particularly problematic given that resource constraints represent the primary barrier to intervention adoption in real-world settings, yet only sixteen percent of studies provided this essential information (Ritzwoller et al., 2009). Most concerning is the near absence of maintenance planning, with fewer than one in ten studies describing sustainability strategies. This finding reflects a well-documented implementation science challenge where interventions demonstrate efficacy during funded research periods but fail to persist once external support ends (Shelton et al., 2018). For health literacy research to achieve its public health potential, the field must fundamentally shift toward prospective implementation planning that addresses sustainability from inception rather than as an afterthought. This will likely require structural changes including journal requirements for implementation reporting, funding mechanisms that support longer follow-up periods, and greater collaboration between researchers and practice settings to ensure interventions are designed with real-world constraints and sustainability in mind from the outset.

### Implications for Policy and Research

This review highlights the importance of developing and implementing culturally and linguistically appropriate, validated measurement tools to evaluate intervention effectiveness. The reliance on nonvalidated or locally adapted tools may limit clinicians' ability to reliably assess patient health literacy and compare outcomes across programs. Standardized metrics would enhance comparability across studies, reduce the delay in research translation, and improve the utility of findings for policymakers. Clinicians and program designers should also consider the specific purpose of measurement when selecting instruments, as commonly used tools such as the NVS or REALM primarily assess functional health literacy, whereas broader instruments may capture multidimensional aspects of health literacy. However, the effectiveness of even the most well-designed patient-focused interventions may be limited without corresponding attention to provider competencies and organizational capacity. Future interventions should systematically incorporate training for health care providers in health literacy principles, including clear communication techniques, Teach-Back methods, and plain language strategies. Beyond individual provider skills, health literacy interventions need to embed organizational health literacy frameworks into intervention design and evaluation. This includes assessing institutional policies, communication systems, navigation support, and structural mechanisms that either facilitate or impede accessible health information delivery. The limited research applying systems thinking and organizational health literacy principles represents a significant gap in the field, as sustainable improvements in health literacy outcomes require alignment across individual, provider, and systems levels. Implementation frameworks such as the Practical, Robust Implementation and Sustainability Model, which underpins the RE-AIM framework, may provide a useful structure for integrating organizational context, stakeholder perspectives, and sustainability considerations into health literacy policy and intervention design. Finally, research on geographically underserved populations—including rural communities and tribal nations—remained limited, underscoring the need for more inclusive and equity-driven approaches to health literacy research.

### Strengths

This review has several strengths. It provides an updated synthesis of U.S.-based health literacy intervention studies conducted over the past decade and offers a detailed examination of the measurement tools used to assess health literacy outcomes. By systematically mapping both validated and nonvalidated instruments across studies, this review highlights ongoing challenges related to measurement consistency and tool selection. In addition, the use of the RE-AIM framework allowed for a structured assessment of how implementation outcomes are reported, providing insight into gaps in adoption, implementation fidelity, cost reporting, and sustainability planning. Together, these contributions extend prior reviews by integrating both measurement practices and implementation considerations, offering practical insights for researchers, practitioners, and policymakers designing and evaluating future health literacy interventions.

### Limitations

This scoping review also has several limitations that should be considered when interpreting the findings. First, the search was limited to a single database (Ovid MEDLINE), which may have led to the exclusion of relevant studies indexed in other databases such as CINAHL, PsycINFO, Scopus, or Embase. This constraint may have introduced publication bias or limited the overall scope. Additionally, formal calibration exercises were not conducted prior to screening, which may have introduced variability in early screening decisions, though discrepancies between reviewers were resolved through discussion or third-party adjudication. As a scoping review, formal risk of bias appraisal of included studies was not conducted, consistent with JBI guidance; readers should interpret the findings accordingly. Also, the review was restricted to English-language publications. While this is a relatively minor limitation given the U.S. focus, it may still under-represent interventions designed for or by linguistically diverse communities, including Spanish-speaking populations. The review excluded gray literature. Although this approach enhances rigor and ensures inclusion of peer-reviewed, high-quality research, it may have led to the omission of relevant community-based or practice-informed interventions, particularly those emerging from nonprofit or public health sectors. There was substantial heterogeneity in the tools used to assess health literacy across studies, which limited comparability and synthesis of findings. In addition, many of the included studies had relatively modest sample sizes (a mean of 202 participants), which may limit statistical power and the generalizability of intervention findings. Finally, variability in how intervention effectiveness was reported across studies hindered our ability to draw deeper conclusions regarding what works best, for whom, and in what contexts.

## Conclusion

This scoping review provides a comprehensive synthesis of U.S.-based health literacy interventions, offering critical insights into the populations served, settings used, measurement practices, and health topics addressed. The findings underscore the field's growth and diversification, with increasing attention to racial and ethnic populations and a wide range of health conditions. However, the review also reveals persistent methodological challenges, including inconsistent use of validated measurement tools, limited inclusion of linguistically diverse populations, and underutilization of implementation science frameworks. By promoting more rigorous, inclusive, and contextually grounded research, the field can better support the development of equitable, effective interventions that improve health outcomes and reduce disparities across the U.S. health care landscape.
